# Efficient In Vitro Plantlet Regeneration from Stolon Explants and Genetic Stability Assessment Using ISSR Markers in the Ornamental Fern *Hypolepis punctata*

**DOI:** 10.3390/plants14162569

**Published:** 2025-08-18

**Authors:** Xinyuan Wang, Xuetong Yan, Keyuan Zheng, Hui Shen, Jianguo Cao, Qiang Zhou, Mulan Zhu

**Affiliations:** 1 Shanghai Key Laboratory of Plant Functional Genomics and Resources, Shanghai Chenshan Botanical Garden, Shanghai 201602, China; xinyuanw109@163.com (X.W.); xty070808@163.com (X.Y.); kyzheng@cemps.ac.cn (K.Z.); shenhui@cemps.ac.cn (H.S.); 2 CAS Center for Excellence in Molecular Plant Sciences, Institute of Plant Physiology and Ecology, Chinese Academy of Sciences, 300 Fenglin Road, Shanghai 200032, China; 3 School of Life Sciences, Jishou University, Jishou 416000, China; zhouqiang@jsu.edu.cn; 4 School of Life Sciences, Shanghai Normal University, Shanghai 200223, China; cao101@shnu.edu.cn

**Keywords:** *Hypolepis punctata*, stolon, green globular bodies (GGBs), in vitro regeneration, genetic fidelity

## Abstract

*Hypolepis punctata*, an aromatic fern with insect-resistant and ornamental potential. Up to date, no studies have reported its micropropagation, particularly using vegetative organs as explants. The optimized stolon sterilization (81.11%) employed 75% ethanol (30 s) and 15% sodium hypochlorite (12 min). The optimal conditions for GGB induction (75.56%) and proliferation (8.46 mm) were achieved using Murashige and Skoog (MS) medium + 2.0 mg/L 6-benzylaminopurine (BA) + 0.2 mg/L 1-naphthaleneacetic acid (NAA). The optimal plant growth regulator (PGR) formula for sporophyte regeneration was 0.5 mg/L BA + 0.1 mg/L NAA + 2 g/L activated charcoal (AC), achieving a 98.89% induction rate and 49.19 buds per explant. The 1/4 MS medium had the greatest promoting effect on biomass accumulation and leaf expansion. Optimal shoot elongation (97.78% success, 4.83 cm) was achieved in 1/4 MS + 0.5 mg/L BA + 0.1 mg/L NAA + 2 g/L AC, and optimized rooting (92.22%) was achieved using 1/4 MS + 0.5 mg/L indole-3-butyric acid (IBA) + 0.1 mg/L NAA + 2 g/L AC, producing 25.27 roots per plantlet. Crucially, ISSR analysis confirmed the genetic stability of all regenerants. This optimized protocol establishes a scalable micropropagation system, enhancing both commercial cultivation and genetic improvement potential in *Hypolepis punctata*.

## 1. Introduction

*Hypolepis punctata* (family Dennstaedtiaceae), is a globally distributed fern species with primary centers of distribution in tropical Asia and tropical America, which thrives in warm, humid, and semi-shaded environment [1,2,3]. Renowned for its elegant fronds, fragrant aroma, and ability to thrive in cultivated environments, this fern holds significant value as an ornamental plant [4,5]. Beyond its aesthetic appeal, *Hypolepis punctata* exhibits notable insect resistance, offering insights into the evolutionary origins of plant–insect interactions and defense mechanisms [5]. Additionally, it holds considerable medicinal potential. In traditional Chinese medicine (TCM), the entire plant of *Hypolepis punctata* is used medicinally, and bioactive compounds extracted from *Hypolepis punctata* exhibit cytotoxic effects against cancer cell lines [6,7,8]. Given its growing market demand, efficient propagation and genetic improvement of *Hypolepis punctata* are of increasing interest.

Currently, fern propagation relies on spore germination [9]. However, challenges exist in spore collection and gametophyte cultivation [10], along with issues such as high variability and poor seedling quality (e.g., weak plant growth), making this method unsuitable for commercial production and high-quality seedling cultivation [11]. Moreover, spore germination is influenced by multiple factors, including light, temperature, mineral salt concentration, and sugar levels. Additionally, due to the asynchronous development of antheridia and archegonia in *Hypolepis punctata* gametophytes, mixed-spore sowing often fails to ensure trait stability. Meanwhile, sporophytes derived from isolated gametophytes through intragametophytic selfing exhibit weaker growth and may even experience sporophyte mortality [4].

Plant tissue culture presents a viable solution for rapid, large-scale propagation while maintaining clonal fidelity [12]. Although tissue culture techniques have been applied to fern species, the field remains in its early developmental stages [13]. To date, no successful tissue culture protocol has been reported for *Hypolepis punctata* in the scientific literature. While spore-based propagation is common in fern tissue culture, challenges, such as microbial contamination due to spore surface complexity [9], low sporophyte conversion rates, and prolonged regeneration timelines, hinder efficiency [14,15]. Alternatively, sporophyte-derived explants may serve as a more reliable sterile material. Compared with callus-mediated [16,17,18] or adventitious bud regeneration systems [11,19], the green globular body (GGB) system offers distinct advantages, including operational simplicity, high regeneration capacity, and efficient whole-plant development [20,21,22]. Moreover, using vegetative organs as explants presents higher regeneration difficulty when compared with spore regeneration, though the regenerated plants exhibit greater genetic stability and can better retain the desirable traits of the superior stock. The induction of green globular bodies (GGBs) is crucial for fern propagation, yet reports on GGB induction in *Hypolepis punctata* remain scarce. To achieve the stable large-scale propagation of *Hypolepis punctata*, it is essential to establish an efficient in vitro regeneration system for this fern species.

In the improvement of plant traits and the study of plant gene functions, the absence of a well-optimized in vitro regeneration protocol remains a major bottleneck [23]. Studies on stable genetic transformation systems in ferns are relatively limited. Bui et al. [24] have reported both transient and stable *Agrobacterium*-mediated transformation methods for the model fern *Ceratopteris richardii* gametophytes. Research by Xiang et al. [25] demonstrates that CRISPR/Cas9 genome editing is feasible in ferns and that PINs (auxin efflux carriers) may play a role in fern leaf development. From a technical perspective, the current limitations in fern genetic transformation primarily stem from challenges such as difficult explant acquisition and low explant differentiation rates, making it difficult to obtain transgenic plants within a short timeframe [26]. Ferns are rarely affected by insects where insect infestation on ferns is 30-fold lower than on flowering plants [27]. However, current research on fern defenses against insects remains scarce compared with seed plants, and our understanding of fern defensive mechanisms is still very limited. Shukla et al. have reported that *Tma12*, a gene derived from the edible fern *Tectaria macrodonta* (Fee) C. Chr., exhibits insecticidal activity against whiteflies (*Bemisia tabaci*) [28]. Huang et al. found that *Hypolepis punctata* can attract insect predators by releasing herbivore-induced volatile organic compounds (HIPVs) [5], future research may identify relevant insect-resistant genes in ferns. By cloning these genes into other plant species, we could develop functional plants with unique pest-resistant characteristics. *Hypolepis punctata* not only exhibits significant ornamental value and strong breeding potential but also harbors robust insect-resistance genes, making it a promising candidate for genetic editing applications in pest control. Plant tissue culture technology can provide abundant experimental materials for such studies and serves as an essential step for both trait improvement and functional gene research in ferns. Therefore, establishing a stable and efficient in vitro regeneration system for *Hypolepis punctata* is critically important.

This study reports an efficient regeneration system for *Hypolepis punctata*, which includes the following critical stages: explant sterilization, GGB induction and proliferation, progressing to sporophyte induction, adventitious shoot elongation, rooting, and acclimatization. Optimizing these stages, this study presents a robust in vitro regeneration protocol capable of producing large quantities of phenotypically uniform *H. punctata* plants. The establishment of this system provides reliable technical support for the large-scale seedling production and genetic improvement of *Hypolepis punctata* and other fern species.

## 2. Results

### 2.1. Preparation of Sterile Materials

**Figure 1:**
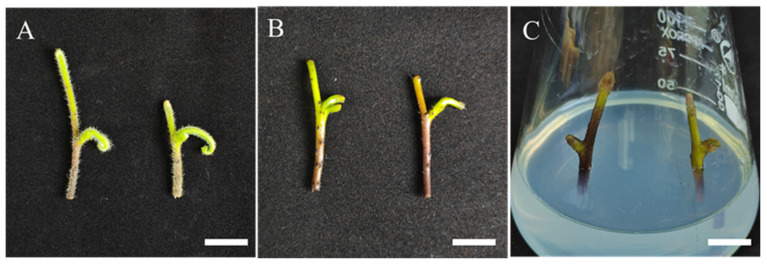
Explant sterilization and bottle inoculation process. (**A**) *Hypolepis punctata* stolons collected from their natural habitat, bar = 2 cm; (**B**) pre-treated *Hypolepis punctata* stolon segments after mechanical cleaning, bar = 1.2 cm; (**C**) Sterilized stolon explants cultured on hormone-free MS medium for 10 days, bar = 1.2 cm.

The pre-treated stolon explants of *Hypolepis punctata* (Figure 1B) were subjected to a combined sterilization treatment using 75% ethanol and 15% sodium hypochlorite (NaClO) to evaluate the effects of sterilization intensity on contamination and survival rates. The results demonstrate that the optimal sterilization protocol consisted of 75% ethanol treatment for 30 s, followed by 15% NaClO for 12 min, achieving a survival rate of 81.11% after 10 days of culture, with the explants exhibiting robust viability (Table 1). While extending the exposure time to ethanol and NaClO effectively reduced contamination rates, excessive sterilization (60 s in 75% ethanol + 15 min in 15% NaClO) led to a significant decline in survival rate (60%), despite lowering the contamination rate to 4.45%. This indicates that prolonged sterilization induces substantial phytotoxicity, resulting in severely compromised explant health, as evidenced by stunted growth and reduced metabolic activity. Such over-sterilized explants exhibited poor performance in subsequent cultures, highlighting the need for balanced sterilization to minimize both contamination and tissue damage.

### 2.2. Effect of MS Macronutrient Intensity on GGB Induction

**Figure 2:**
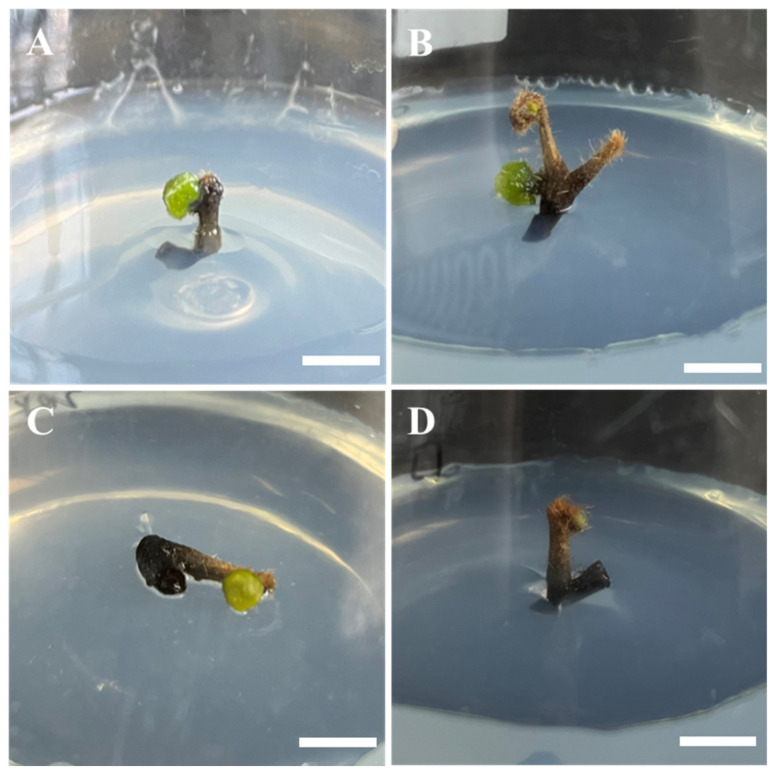
The effect of MS macronutrient intensity on GGB induction. (**A**) Induced GGB formation on 2 MS medium with 1 mg/L BA + 0.1 mg/L NAA, bar = 0.5 cm; (**B**) induced GGB formation on MS medium with 1 mg/L BA + 0.1 mg/L NAA, bar = 0.5 cm; (**C**) induced GGB formation on 1/2 MS medium with 1 mg/L BA + 0.1 mg/L NAA, bar = 0.5 cm; (**D**) induced GGB formation on 1/4 MS medium with 1 mg/L BA + 0.1 mg/L NAA, bar = 0.5 cm.

Distinct Murashige and Skoog (MS) [29] macronutrient intensities on GGB induction were investigated. Among the four basal MS media with varying mineral salt concentrations (2 MS, MS, 1/2 MS, and 1/4 MS), all could induce GGB formation, though significant differences were observed in induction rates and GGB status (Table 2). GGBs induced in MS and 1/2 MS media exhibited healthy green coloration and robust morphology (Figure 2B,C), while those in 2 MS medium showed chlorosis and weak growth (Figure 2C). GGBs induced in 1/4 MS medium eventually browned and died (Figure 2D). Quantitative analysis revealed that full-strength MS medium achieved the highest induction rate (54.00%), significantly surpassing 1/2 MS medium (36.67%). This study demonstrates that standard MS medium is optimal for GGB induction in *Hypolepis punctata*, with 1/2 MS medium serving as a viable alternative. The GGBs induced in these two media displayed superior morphological characteristics compared with other concentrations. The superiority of MS medium was manifested in both quality and quantity of induced GGBs, indicating that its optimized nutrient composition better supports the initial stages of GGB formation and development in this fern species.

### 2.3. Effect of the Combination of BA and NAA on the Induction and Proliferation of Green Globular Bodies

Plant growth regulators (PGRs) play a crucial role in in vitro plant regeneration, the effects of cytokinin (BA) and auxin (NAA) on GGB induction and proliferation in *Hypolepis punctata* depend on their specific concentration combinations. Notably, no GGBs were induced in the hormone-free control group, indicating that GGB formation and proliferation strictly depend on PGRs. Significant differences in GGB induction and proliferation were observed among MS media supplemented with different PGR combinations (Table 3). At a low BA concentration (0.2 mg/L), the induction rate was extremely low, and the GGB diameter was small. In contrast, the combination of 2.0 mg/L BA and 0.2 mg/L NAA achieved the highest induction rate (75.56%), with an average GGB diameter of 8.46 mm. The results demonstrate that NAA must reach a threshold concentration (0.2 mg/L) to effectively promote GGB induction and proliferation. When combined with 1, 2, and 3 mg/L BA, higher NAA concentrations (0.2 mg/L) significantly enhanced induction and proliferation compared with lower concentrations (0.01 mg/L).

### 2.4. Effect of PGRs on Sporophyte Regeneration

To determine the optimal plant growth regulator (PGR) combination for adventitious shoot induction, all GGBs were transferred onto 1/2 MS media containing different concentrations of BA, NAA, and activated charcoal (AC).

**Figure 3:**
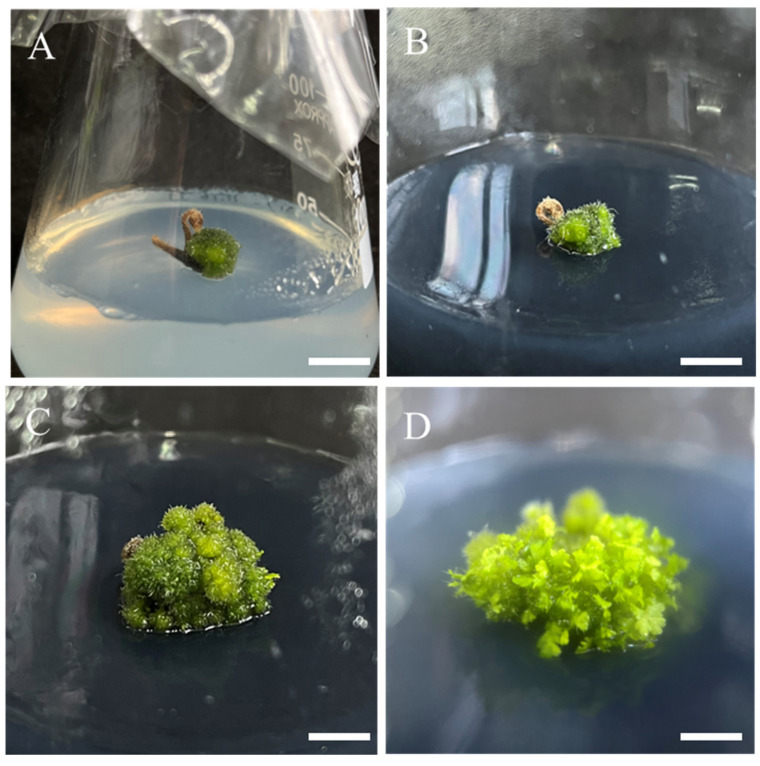
Sporophyte induction process from GGBs of *Hypolepis punctata*. (**A**) GGB proliferated on MS medium with 2 mg/L BA + 0.2 mg/L NAA, bar = 1 cm; (**B**) GGBs at differentiation stage I: 1-week culture on MS medium with 0.5 mg/L BA + 0.1 mg/L NAA + 2 g/L AC, bar = 0.9 cm; (**C**) GGBs at differentiation stage II: 3-week culture on MS medium with 0.5 mg/L BA + 0.1 mg/L NAA + 2 g/L AC, bar = 0.5 cm; (**D**) highly synchronized sporophytes induced from GGBs: 6-week culture on MS medium with 0.5 mg/L BA + 0.1 mg/L NAA + 2 g/L AC, bar = 0.4 cm.

Compared with the optimal medium for GGB proliferation, lower concentrations of BA were more conducive to sporophyte induction from GGBs (Table 4). The cytokinin (BA) concentration played a decisive role in sporophyte induction. When the BA concentration exceeded 0.5 mg/L, GGBs tended to continue proliferating, resulting in a significant decrease in sporophyte induction rate. Moreover, excessively high NAA concentrations slightly inhibited shoot induction—when maintaining constant BA levels, increasing NAA to 0.5 mg/L led to reductions in both induction rate and adventitious bud number. Additionally, under identical PGR concentrations, the incorporation of AC simultaneously enhanced both induction rate and bud number, demonstrating AC’s significant promotive effect on *Hypolepis punctata* growth. After 8 weeks of culture, the medium containing 2 g/L AC, 0.5 mg/L BA, and 0.1 mg/L NAA achieved the highest GGB differentiation frequency (98.89%) (Figure 3D), producing an average of 49.19 healthy green adventitious bus per explant, thereby establishing this formulation as the optimal medium composition for GGB differentiation.

### 2.5. Effect of Basic Culture Medium on Leaf Expansion and Biomass Accumulation of Sporophytes

**Figure 4:**
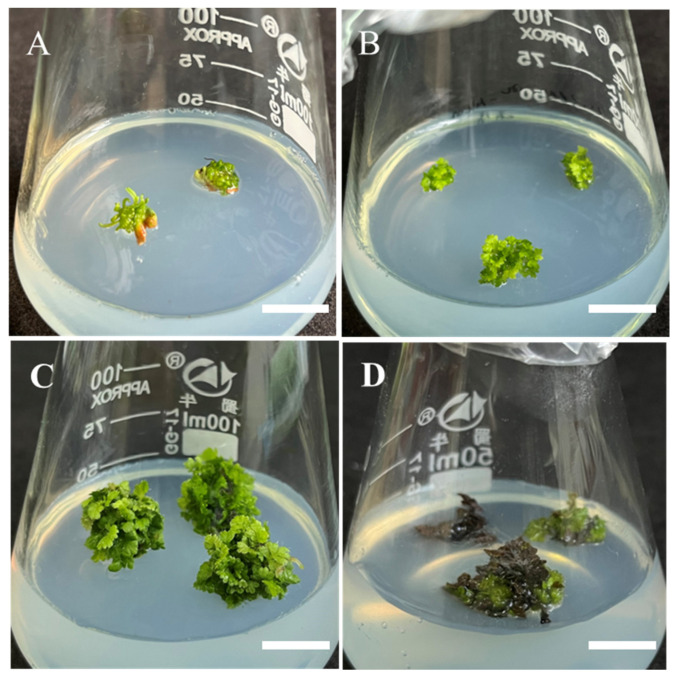
The effect of the basal medium on leaf expansion and biomass accumulation. (**A**) Regenerated plantlets on N6 medium with 0.5 mg/L BA + 0.05 mg/L NAA, bar = 2 cm; (**B**) regenerated plantlets on 1/2 MS medium with 0.5 mg/L BA + 0.05 mg/L NAA, bar = 2 cm; (**C**) regenerated plantlets on 1/4 MS medium with 0.5 mg/L BA + 0.05 mg/L NAA, bar = 2 cm; (**D**) regenerated plantlets on DCR medium with 0.5 mg/L BA + 0.05 mg/L NAA, bar = 2 cm.

**Figure 5:**
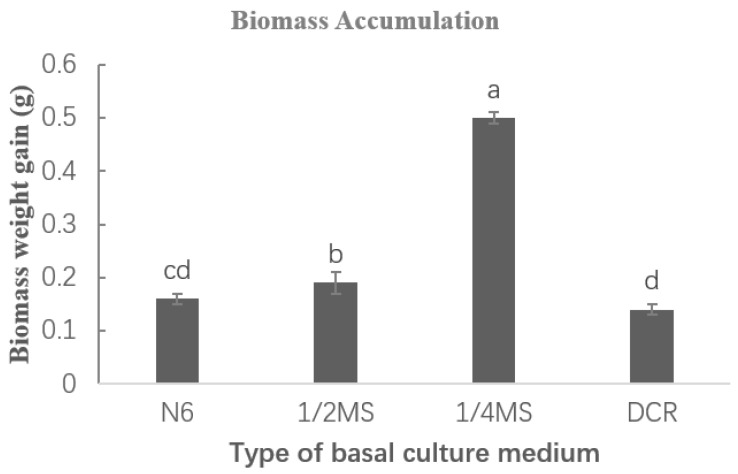
Biomass amplification under different culture media. The vertical axis (*Y*-axis) represents the biomass increment per individual plant (g), while the horizontal axis (*X*-axis) indicates the basal medium types used for different treatment groups. Different letters indicate significant differences (*p* < 0.05).

The sporophytes of *Hypolepis punctata* with well-developed leaf morphology were segmented and transferred to different basal media for adventitious shoot leaf expansion and biomass accumulation induction, with each medium supplemented with 0.5 mg/L BA and 0.05 mg/L NAA. As shown in Figure 4, both 1/2 MS and 1/4 MS media produced vigorous green leaves with significant expansion, though the degree of expansion in 1/2 MS medium was slightly less pronounced than in 1/4 MS. When cultured on Chu’s N6 (N6) medium [30], the explants showed chlorosis in leaves along with partial vitrification. (Figure 4A). When cultured on Douglas-fir Cotyledon Revised (DCR) [31] medium, the explants showed chlorosis and necrosis (Figure 4D). Regarding biomass accumulation, comprehensive results demonstrated that 1/4 MS basal medium combined the highest biomass accumulation capacity (Figure 5) with optimal morphological development (vigorous growth and maximum leaf expansion), representing the most suitable formulation for adventitious shoot leaf expansion and biomass accumulation in *Hypolepis punctata*. This finding suggest that moderately reduced nutrient levels (1/4 MS) better support developmental processes.

### 2.6. Effects of BA and AC on Shoot Elongation and Growth

**Figure 6:**
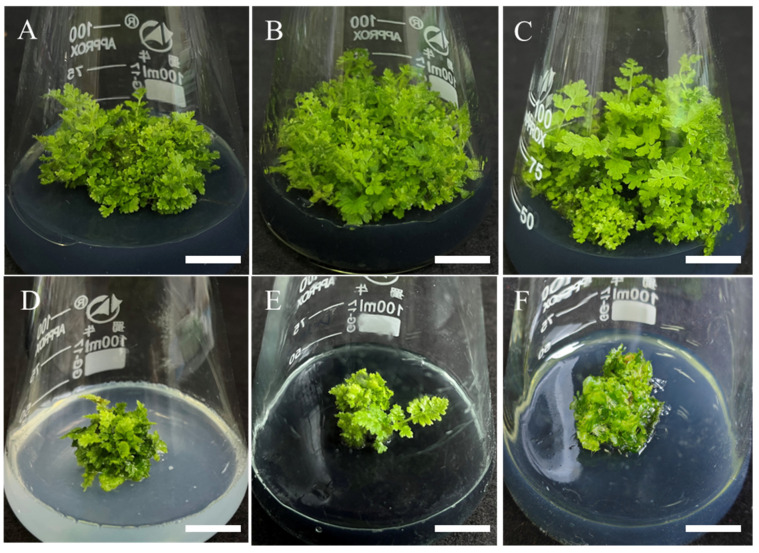
Effects of BA and AC on *Hypolepis punctata*. elongation and growth conditions. (**A**) Leaf enlargement regenerated plantlets with 0.1 mg/L BA, 2 g/L AC, 0.1 mg/L NAA, bar = 1.7 cm; (**B**) leaf enlargement regenerated plantlets with 1.0 mg/L BA, 2 g/L AC, 0.1 mg/L NAA, bar = 1.7 cm; (**C**) leaf enlargement regenerated plantlets with 0.5 mg/L BA, 2 g/L AC, 0.1 mg/L NAA, bar = 2 cm; (**D**) leaf enlargement regenerated plantlets with 0.1 mg/L BA, 0.1 mg/L NAA, bar = 2 cm; (**E**) leaf blades of unenlarged regenerated plantlets with 0.5 mg/L BA, 2 g/L AC, 0.1 mg/L NAA, bar = 1.7 cm; (**F**) leaf blades of unenlarged regenerated plantlets with 1 mg/L BA, 2 g/L AC, 0.1 mg/L NAA, bar = 1.7 cm.

To promote shoot elongation, adventitious buds were transferred to 1/4 MS medium supplemented with varying concentrations of BA, AC, and 0.1 mg/L NAA. As shown in Table 5, BA concentration significantly affected shoot elongation in *Hypolepis punctata*. The 0.1 mg/L BA treatment group showed an elongation rate of 54.44% with an average shoot height of 2.3 cm, producing healthy green leaves (Figure 6A). Optimal results were achieved with 0.5 mg/L BA, yielding 97.78% elongation rate and maximum average shoot height (4.83 cm), with plants exhibiting well-expanded, vibrant green leaves and uniform elongation morphology (Figure 6C). The elongation rates followed a normal distribution curve, declining when BA concentrations exceeded 0.5 mg/L. The 1.0 mg/L treatment group showed significantly reduced plant height compared with the 0.5 mg/L group, with smaller and more crowded leaves (Figure 6B). AC supplementation significantly enhanced both elongation rate and shoot height. Cultures in AC-free medium produced notably shorter plants (Figure 6D) with significantly lower elongation rates than AC-supplemented groups. Notably, when directly transferred to elongation medium supplemented with AC, adventitious shoots that lacked prior cultivation with expanded fronds either failed to elongate (Figure 6E) or exhibited markedly reduced elongation rates with asynchronous growth patterns (Figure 6F). These results demonstrate that the optimal combination for synchronized adventitious bud elongation in *Hypolepis punctata* is 1/2 MS medium supplemented with 0.5 mg/L BA, 0.1 mg/L NAA, and 0.2 g/L AC. Furthermore, the leaf expansion culture stage is essential for successful shoot elongation in this fern species.

### 2.7. Effect of IBA on Rooting

**Figure 7:**
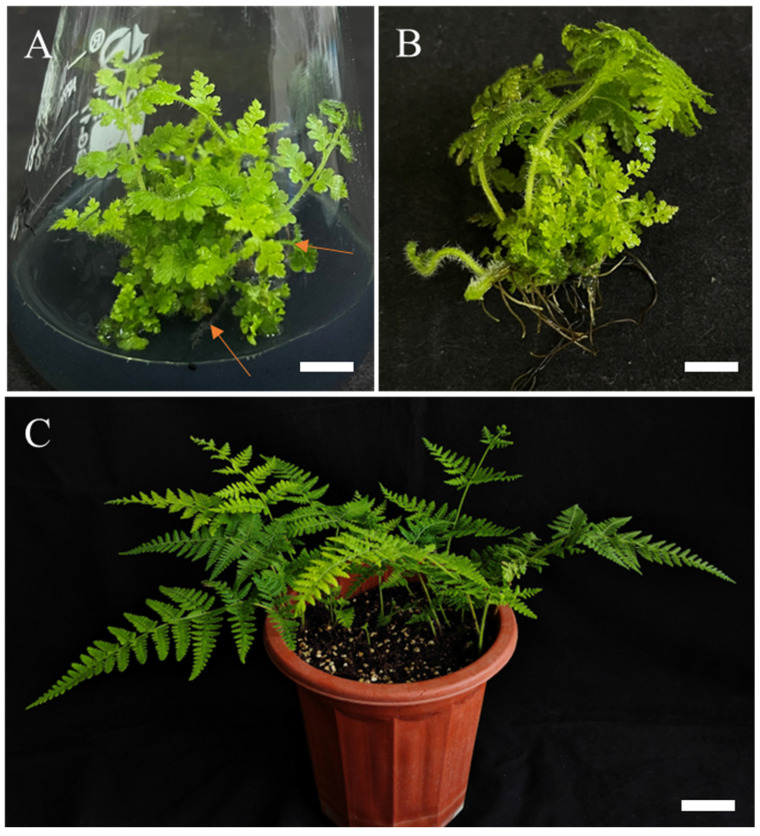
Rooting and acclimatization of *Hypolepis punctata* regenerated shoots. (**A**) Rooted plantlets after 6-week culture on 1/4 MS medium with 0.5 mg/L IBA + 0.1 mg/L NAA + 2 g/L AC, the arrow marks/signifies the root, bar = 1.5 cm. (**B**) Regenerated plantlets harvested after 8 weeks of shoot regeneration culture, bar = 1.5 cm. (**C**) Plants acclimatized for 8 months, bar = 5 cm.

As shown in Table 6, the combination of NAA and Indole-3-butyric acid (IBA) significantly outperformed NAA alone in root induction efficiency. When NAA was combined with low concentrations of IBA (0.1 mg/L), both treatments exhibited low rooting rates and prolonged root primordium formation cycles. Within a certain concentration range, the rooting rate initially increased rapidly and then slightly decreased with increasing IBA concentrations. The highest rooting rate (98.52%) was achieved after 21 days of culture at 0.7 mg/L IBA, though the average root number (18.91 roots) was lower than that obtained with 0.5 mg/L IBA (25.27 roots) (Figure 7B). This optimized auxin combination not only improved rooting efficiency but also maintained the regenerative capacity of tissue-cultured plantlets (Figure 7C).

### 2.8. Genetic Fidelity Assessment

**Figure 8:**
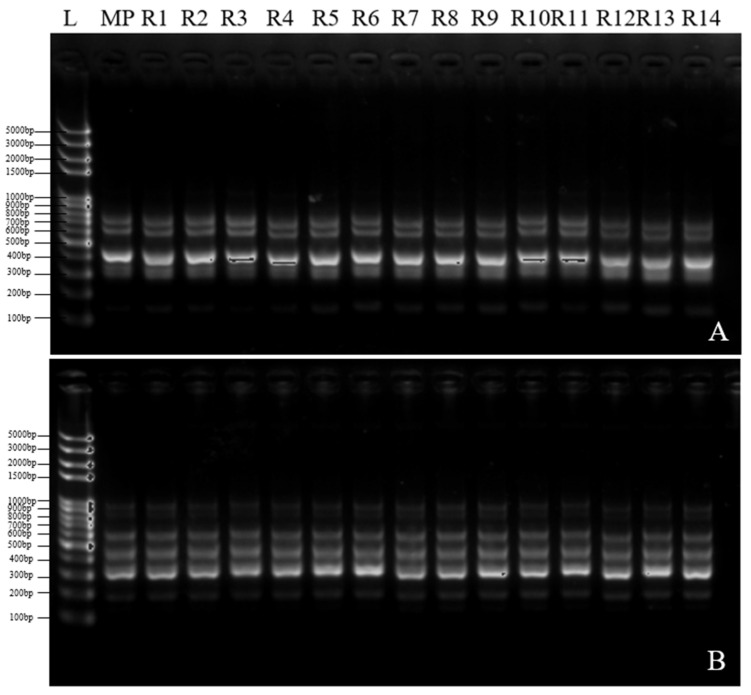
Genetic stability assessment of *Hypolepis punctata* using ISSR markers and genetic fidelity of regenerated plants, determined using ISSR molecular markers. Profiles were obtained from primers ISSR14 (**A**) and ISSR11 (**B**). Lane M: molecular marker (100 bp–5 kb). MP: mother plant. Lanes R1–14: regenerated plants.

A total of 14 regenerated plants were taken, with the mother plant as the control, to evaluate genetic fidelity using 25 ISSR primers. Finally, 10 ISSR primers were selected, generating a total of 55 distinct monomorphic bands for subsequent PCR amplification. The banding patterns ranged from 3 (ISSR15) to 8 (ISSR6), with band sizes varying from 100 to 1000 bp (Table 7). Figure 8 displays the banding patterns of ISSR14 and ISSR11. In summary, the results of this study indicate that no genetic variation occurred in the regenerated plants at the molecular level.

## 3. Discussion

This study aims to develop an efficient in vitro regeneration protocol for *Hypolepis punctata* using the GGB system. In this study, the mineral salt concentration of the culture medium, the hormone combinations, and the addition of activated charcoal were identified as key factors influencing GGB induction, sporophyte formation, and young plant regeneration. By optimizing various parameters, we have developed an efficient tissue culture system for the ornamental fern *Hypolepis punctata*, thereby establishing a technical foundation for large-scale propagation of high-quality shoots, genetic engineering, gene editing, and germplasm conservation.

The selection of basal medium is a critical factor in the induction, proliferation, and sporophyte regeneration of green globular bodies (GGBs) [32]. Our results indicate that MS basal medium is relatively suitable for GGB induction in *Hypolepis punctata*. High concentrations of macronutrients cause the induced GGBs to turn yellow, hindering subsequent growth (Table 1, Figure 2A), likely due to osmotic stress from high salinity or ion imbalance. Conversely, excessively low macronutrient levels make GGB induction difficult, and the induced GGBs exhibit poor viability (Table 1, Figure 2D), while a low-salt-induced nutrient limitation may restrict growth. Future research should analyze physiological determinants of observed growth variations and induction efficiency. Different types of ferns require distinct basal media for optimal growth [33]. This study compared the effects of N6, DCR, 1/2 MS, and 1/4 MS basal media on the leaf development of *Hypolepis punctata* sporophytes, with the best growth observed in 1/4 MS medium. By analyzing the composition of these four media, we hypothesize that higher concentrations of nitrate nitrogen (NO_3_^−^) in the basal medium may inhibit normal sporophyte development in *Hypolepis punctata*, leading to impaired leaf morphogenesis. In addition, excessively high levels of Ca^2+^ and Mg^2+^ may exert toxic effects, adversely affecting the growth and development of Hypolepis punctata and potentially leading to plant mortality. This observation aligns with the findings of Mikuła et al. [34] on *Cyathea delgadii*, where inappropriate mineral salt concentrations increased the frequency of abnormal regenerated plantlets. Moreover, the optimal basal medium varies among fern species. Yu et al. [33] have reported that approximately 1/4 MS was the best mineral salt concentration for GGB differentiation and young sporophyte growth in *Pteris aspericaulis*. Conversely, Fernandez et al. [35] found that MS medium promoted sporophyte development in *Lastrea affinis* ssp. affinis. Additionally, Thakur et al. have demonstrated that 1/4 MS supplemented with 1.0% activated charcoal (AC) facilitated meristematic nodule (MN)-based plant regeneration as well as root and leaf development in *Matteuccia struthiopteris* [36].

Plant growth regulators (PGRs) are a key factor in plant tissue culture experiments for GGB induction. In this study, GGBs were induced in all experimental groups except the PGR-free control group. Similar to observations in *Asplenium nidus* [37], *Cibotium barometz* [38], and *Alsophila costularis* [14], GGB induction in *Hypolepis punctata* was highly dependent on the presence of cytokinin, and the induction frequency increased with higher cytokinin concentrations. However, cytokinins also exhibit a dual role, as some studies have reported their inhibitory effect on bud proliferation [37,39,40]. In this study, when the BA concentration reached 1 mg/L, adventitious bud proliferation in *Hypolepis punctata* was suppressed, whereas lower BA concentrations were more beneficial for induction (Table 4), confirming the cytokinin-mediated regulation of GGB organogenesis first proposed by Higuchi et al. [37]. Furthermore, in fern tissue culture, the combination of cytokinins and auxins resulted in higher GGB induction rates compared with the use of cytokinins alone. For instance, in *Platycerium bifurcatum*, the combination of cytokinins (BA and TDZ) with auxin (NAA) led to higher GGB induction rates than the use of single cytokinin or auxins [22]. These findings are consistent with our results, where higher NAA concentrations (0.2 mg/L) combined with 1–3 mg/L BA significantly improved GGB induction and proliferation compared with lower NAA concentrations (0.01 mg/L), further supporting the superiority of cytokinin-auxin combinations over single PGRs in GGB induction.

Activated charcoal (AC) exerts significant effects on sporophyte induction and juvenile plant regeneration in ferns. Characterized by a microporous network and high surface area, it exhibits remarkable adsorption capabilities [41]. AC is known to adsorb toxic metabolites, phenolic compounds, and PGRs secreted by explants, thereby promoting morphogenesis, embryogenesis, and root growth in plant cultures [42]. It also influenced morphogenesis, cell division, and sex determination in gametophytes of Equisetum arvense and Blechnum spicant [43]. The effect of AC supplementation varies significantly across different fern species. In the case of *Platycerium bifurcatum*, AC addition significantly inhibited sporophyte development from GGBs [22]. Conversely, AC had a positive effect on sporophyte regeneration in *Platycerium bifurcatum* [44] and *Lemmaphyllum microphyllum* C. Presl [45]. In this study, AC supplementation enhanced both the induction rate during sporophyte induction and the number of adventitious shoots (Table 4), while significantly improving the elongation rate and shoot length of *Hypolepis punctata* adventitious buds (Table 5). Additionally, studies have shown that AC promotes root formation in ferns [11]. Therefore, AC was added to the elongation and rooting media in this study to enhance root development.

This study provides the first evidence that plants subjected to frond expansion cultivation exhibit superior performance during subsequent elongation and rooting phases (Figure 4). In *Hypolepis punctata*, the regenerated plantlets demonstrated remarkable rooting and acclimatization efficiency, achieving 97–100% survival rates. This high viability likely stems from their uniform shoot elongation, which showed strong correlation with prior frond expansion. Notably, as demonstrated in woody plants [40,46], robust plantlets capable of resisting exogenous hormone depletion form the foundation for successful rooting, underscoring the critical importance of obtaining uniformly elongated plantlets.

Maintaining genetic integrity is one of the most critical factors determining the success of in vitro propagation. Traditional methods for detecting genetic variation primarily rely on morphological analysis. However, as many genetic variations do not necessarily manifest phenotypically, accurate detection based solely on morphology is challenging. Recently, ISSR markers have been widely used to assess the genetic stability of regenerated plants [47]. In this study, ISSR analysis confirmed the genetic fidelity between regenerated *Hypolepis punctata* plants and the mother plant, demonstrating that the regenerants were “true-to-type,” with comparable banding patterns. While establishing an in vitro regeneration system via plant rhizomes presents significant challenges [11], this study successfully achieved genetically stable plant regeneration by utilizing stolons—rather than spores—as the primary explant source. Maintaining the superior characteristics of elite cultivars is especially critical in ornamental plants. This finding offers a valuable framework for applying stolon-based explants to ensure genetic stability in the micropropagation of other fern species.

## 4. Materials and Methods

### 4.1. Plant Material and Sterilization

The experiment utilized stolons of *Hypolepis punctata* with superior phenotypes as explants, which were collected from wild populations in Sangzhi County, Hunan Province and subsequently introduced to the Shanghai Chenshan Botanical Garden. A pretreatment protocol was implemented on field-collected stolon explants of *Hypolepis punctata* before initiating standard sterilization protocols. First, the stolons were cut into 5–7 cm segments, gently scrubbed with moist gauze soaked in soapy water to remove surface hairs, and rinsed under running water for 1 h. The explants were then subjected to ultrasonic treatment (40 kHz) (Xiaomei Ultrasonic Instruments (Kunshan) Co., Ltd., Kunshan, China) in a 2‰ potassium permanganate solution for 10 min, followed by a final 1 h rinse under running water to complete the pretreatment procedure. The pre-treated explants were surface-sterilized with 75% (*v*/*v*) ethanol (Shanghai Titan Technology Co., Ltd., Shanghai, China) for 30–60 s, followed by treatment with 15% (*v*/*v*) sodium hypochlorite (Shanghai Titan Technology Co., Ltd., Shanghai, China) for 8, 12, and 15 min, and washed five times with aseptic water. The sterilized explants were then inoculated onto MS medium, with 1–2 explants per bottle, totaling 30 explants and 3 replicates. Following a 10-day culture period, explant viability and contamination incidence were quantitatively evaluated. After decontamination, plants were trimmed under sterile conditions for downstream experiments. The contamination percentage was calculated by dividing the number of contaminated explants by the total number of explants, while the survival rate was determined by dividing the number of viable explants by the total explant count.

All plant culture media used in this study were supplemented with 30 g/L sucrose (Xilong Scientific Co., Ltd., Shantou, China) and 5 g/L agar (Shanghai Shize Biotechnology Co., Ltd., Shanghai, China), with the pH of the basal medium adjusted to 5.8. The media were sterilized in an autoclave (SHENAN, Shanghai, China) at 0.105 MPa pressure and 121 °C for 20 min. The cultures were cultivated under light conditions, using an LED lamp as the light source, at 25 ± 2 °C with a 16 h photoperiod and a light intensity of 42 μmol/m^2^/s.

### 4.2. Induction of Green Globular Bodies

The effect of macronutrient concentration in the basal medium on green globular body (GGB) induction was first investigated. To investigate the effects of MS medium with varying macronutrient concentrations on GGB induction, sterile explants were cultured on 2 MS, MS, 1/2 MS, and 1/4 MS media (where n MS denotes an MS basal medium with macronutrients adjusted to n-fold the standard MS concentration, while maintaining original iron and micronutrient levels). Each medium was supplemented with 1 mg/L 6-benzylaminopurine (BA) and 0.1 mg/L NAA. Subsequently, to determine the optimal concentrations of BA and NAA combinations for GGB induction, sterile explants were inoculated onto MS media supplemented with varying concentrations of BA (0.2, 1, 2, and 3 mg/L) and NAA (0.01 or 0.2 mg/L). GGB induction efficiency was evaluated after 6 weeks of cultivation. Each treatment consisted of 30 explants, with one explant per container (100 mL conical flask), and was replicated three times. The growth chamber maintained consistent environmental parameters as previously specified. GGB induction efficiency was quantified by determining the percentage of explants that developed GGBs relative to the total cultured explants.

### 4.3. Proliferation of Green Globular Bodies

Based on the GGB induction assessment, we further evaluated the effects of six different BA (0.2, 1, 2, and 3 mg/L) and NAA (0.01 or 0.2 mg/L) combinations on GGB proliferation. GGBs that had grown to approximately 2 mm in diameter were sub-cultured back onto their original induction media. After 6 weeks of culture, the final diameter of GGBs was measured to assess proliferation effects. Each treatment involved inoculating 30 GGBs (with one GGB per 100 mL triangular glass bottle) and was conducted with 3 replicates.

### 4.4. Regeneration of Sporophytes

To induce sporophyte regeneration from GGBs and obtain adventitious bud clusters, produced GGBs (approximately 7 mm in diameter) were placed in 100 mL Erlenmeyer flasks containing 1/2 MS basal medium supplemented with varying concentrations of BA (0.1–1.5 mg/L), NAA (0.05, 0.1, 0.2, and 0.5 mg/L), and activated charcoal (AC; 0 or 0.2 g/L). Each experimental treatment consisted of 3 replicates, with 30 explants per trial. The growth chamber conditions followed established protocols. Following a 6-week cultivation period, shoot regeneration was quantified by counting newly formed shoots and calculating sporophyte regeneration efficiency. The sporophyte induction rate was calculated as the percentage of explants that developed sporophytes relative to the total number of cultured explants.

### 4.5. Leaf Expansion and Biomass Accumulation of Sporophytes

The impact of different types of basic media on leaf expansion and biomass amplification was carried out by placing adventitious buds in four distinct basal media with varying nutrient levels (N6, 1/2 MS, 1/4 MS, and DCR) with 0.5 mg/L BA and 0.05 mg/L NAA. After 4 weeks of culture, leaf morphology was observed and fresh weight increment was measured to evaluate biomass changes. Each treatment consisted of 30 explants (one explant per 100 mL Erlenmeyer flask) with 3 replicates.

### 4.6. Elongation of Adventitious Shoots

To investigate the hormonal regulation during adventitious shoot elongation. Adventitious shoots generated from leaf-expansion cultures were aseptically transferred to elongation media containing 1/4 MS basal salts supplemented with 0.1 mg/L NAA in combination with varying concentrations of BA (0.1, 0.5, and 1.0 mg/L) and AC (0 or 0.2 g/L). This factorial experimental design comprised 30 replicate explants per treatment condition, with individual shoots cultured in separate 100 mL Erlenmeyer flasks. Following an initial 4-week culture period, including one subculture cycle, morphometric parameters were evaluated after 8 weeks. Quantitative analysis included determination of both the shoot elongation rate (calculated as the percentage of shoots exceeding 2 cm in height relative to the total number of cultured shoots) and mean shoot height per explant.

### 4.7. Rooting and Acclimatization

The study employed a systematic approach to investigate root induction in adventitious shoots by transferring well-developed elongated shoots to a rooting medium composed of 1/4 MS basal salts supplemented with a gradient of indole-3-butyric acid (IBA) concentrations (0–1.0 mg/L) and 0.1 mg/L NAA. The experimental design featured 30 biological replicates per IBA treatment level, with individual explants cultured in 100 mL triangular glass vessels, and was conducted with three technical repetitions. During the 4-week subculture cycles, root development parameters were quantitatively assessed, including both root number per explant and rooting efficiency, the latter calculated as the percentage of successfully rooted plantlets relative to the initial number of cultured shoots (root induction rate = [rooted plantlets/total shoots] × 100%).

During acclimatization, healthy regenerated plantlets exhibiting robust root systems and vigorous growth were chosen for transplantation.

The acclimation process involved the following: (1) The container lids were loosened and an appropriate amount of sterile water was added, followed by a 3-day acclimation period in shaded conditions to facilitate initial environmental adaptation; (2) the plantlets were then removed and rinsed under running water to eliminate agar residues from the roots [48]; (3) we transferred the plantlets to nursery pots with a 9.8 cm internal diameter and 8.2 cm vertical dimension and with a substrate composition of peat (Klasman), perlite (Tasha), vermiculite (Viagrow) and coco coir (Viagrow) at a respective ratio of 3:1:1:1 (*v*/*v*). Transparent plastic bags were used to cover the nursery pots to preserve humidity under acclimatization at a 16/8 h light/dark cycle; the growth chamber operated with LED lighting at 42 mmol/m^2^/s, and the environment temperature was maintained at 25 ± 3 °C. After 2 weeks, as the plantlets adapted, the plastic covers were progressively taken off. Plant survival rates were assessed 6 weeks later. The regenerated plants were then transplanted into larger propagation pots (18.6 cm diameter × 16.2 cm height) when stable growth is established and plant size necessitates.

### 4.8. Genetic Homogeneity Analysis

To evaluate genetic stability, 14 healthy regenerated plants that had been sub-cultured for over 10 generations, along with the mother plant, were randomly selected as experimental materials.

Fresh leaves were immediately flash-frozen in liquid nitrogen upon collection, and genomic DNA was extracted from *Hypolepis punctata* using a Plant Genomic DNA Kit (Tiangen Biotech, Beijing, China) following the manufacturer’s protocol [49]. The quality and purity of the extracted DNA were assessed using a NanoDrop™ spectrophotometer (Thermo Fisher Scientific, Waltham, MA, USA). Twenty-five ISSR random primers from Sangon Biotech (Shanghai, China) were screened, and those producing multiple amplification bands were selected for DNA amplification. The 20 μL PCR reaction system consisted of 10 μL 2× Taq PCR Master Mix, 0.5 mmol primers (Sangon Biotech, Shanghai, China), and 100 ng genomic DNA. Amplification was performed in a thermal cycler (Eppendorf Mastercycler^®^ EP Gradient S, Hamburg, Germany) with the following program: initial denaturation at 95 °C for 5 min; 35 cycles of denaturation at 95 °C for 30 s, annealing at 58 °C for 30 s, and extension at 72 °C for 90 s; followed by a final extension at 72 °C for 10 min and storage at 16 °C. The amplification products were electrophoretically separated on a GelRed-stained 1.5% agarose gel (1× TAE buffer) and visualized under UV light using a gel documentation system (Bio-Rad, Hercules, CA, USA). Product sizes were estimated by comparison with a 100 bp Plus II DNA Ladder (Vazyme, Nanjing, China), with clear and distinct DNA bands being prioritized for analysis.

### 4.9. Statistical Analysis

The experimental data were analyzed using the SPSS 27 statistical software package (IBM, Armonk, NY, USA). Following one-way analysis of variance (ANOVA), Duncan’s multiple range test was employed to compare significant differences among treatment groups when significant variations were detected (*p* < 0.05).

## 5. Conclusions

**Figure 9:**
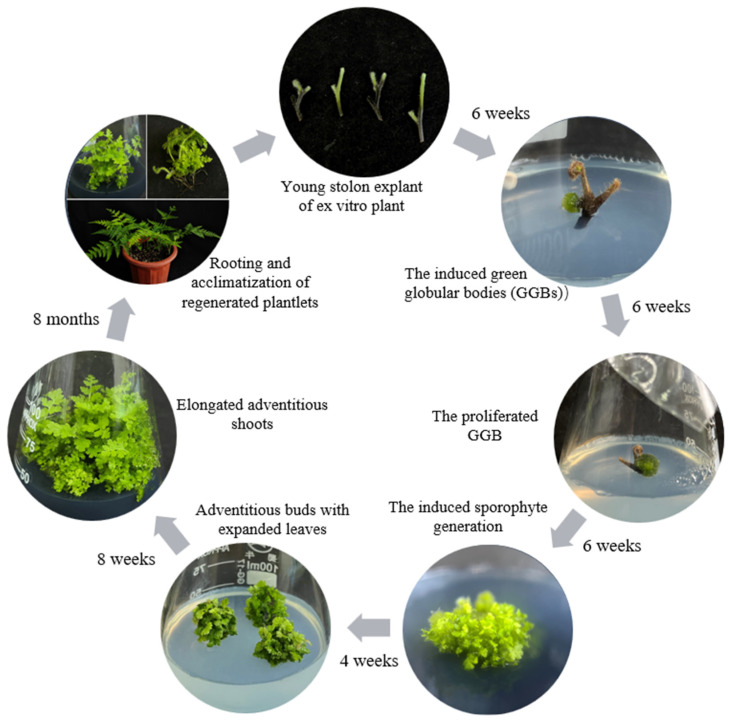
Flowchart of the efficient regeneration system for *Hypolepis punctata*. Based on the above results, a scheme for the efficient regeneration of GGB systems of *Hypolepis punctata* using stolon explants was developed.

This study developed an optimized in vitro regeneration protocol for *Hypolepis punctata* utilizing stolon explants (Figure 9), achieving a remarkable propagation coefficient of 49.11 per explant through adventitious shoot induction, thereby establishing a rapid and season-independent production system, which may be suitable for other fern species to some extent. The key findings demonstrate that green globular body (GGB) induction and proliferation were optimally regulated by phytohormonal synergy, with MS medium containing 2.0 mg/L BA and 0.2 mg/L NAA yielding maximal efficiency. Shoot elongation was highly dependent on BA concentration (optimal at 0.5 mg/L) and activated charcoal supplementation (2 g/L). Meanwhile, synchronous shoot development required prior expanded leaf culture on 1/4 MS medium, as sporophytes without leaf expansion culture exhibited asynchronous elongation or failed to elongate. The rhizogenesis phase was most effectively induced on 1/4 MS medium supplemented with 0.5 mg/L IBA. Importantly, ISSR marker analysis verified the complete genetic stability of regenerated plantlets compared with the donor mother plant, confirming the reliability of this micropropagation system for commercial-scale production of true-to-type *Hypolepis punctata*.

## Figures and Tables

**Table 1:** Effects of disinfection treatments on the contamination rate and survival rate of explants.

75% Ethanol (s)	15% Sodium Hypochlorite (min)	Contamination Rate (%)	Survival Rate (%)
30	8	48.89 ± 2.22 d	51.11 ± 2.22 d
30	12	17.78 ± 1.11 b	81.11 ± 1.11 a
30	15	12.22 ± 2.94 b	70.00 ± 1.92 b
60	8	33.33 ± 1.93 c	57.78 ± 1.11 cd
60	12	14.44 ± 2.94 b	67.78 ± 2.94 b
60	15	4.45 ± 2.22 a	60.00 ± 3.33 c

Data represent mean ± standard error. Duncan’s various lowercase letters show significant differences between the data at the 5% level, using the multiple range test at *p* < 0.05.

**Table 2:** Effect of MS macronutrient intensity on the induction of green globular bodies in *Hypolepis punctata*.

MS Macronutrient Intensity	GGB Induction Rate (%)
2 MS	30.00 ± 2.31 c
MS	54.00 ± 1.15 a
1/2 MS	36.67 ± 1.76 b
1/4 MS	18.67 ± 0.67 d

Data represent mean ± standard error. Duncan’s various lowercase letters show significant differences between the data at the 5% level, using the multiple range test at *p* < 0.05.

**Table 3:** The effect of the combination of BA and NAA on the induction and proliferation of green globular bodies.

BA (mg/L)	NAA (mg/L)	GGB Induction Rate (%)	GGB Diameter (mm)
0	0	0.00	——
0.2	0.2	16.67 ± 1.93 e	5.14 ± 0.14 e
1	0.2	52.23 ± 2.22 c	6.11 ± 0.18 d
2	0.01	24.44 ± 1.11 e	7.97 ± 0.16 b
2	0.2	75.56 ± 2.93 a	8.46 ± 0.12 a
3	0.01	45.53 ± 2.22 d	7.31 ± 0.17 c
3	0.2	67.78 ± 1.11 b	8.40 ± 0.09 ab

Data represent mean ± standard error. Duncan’s various lowercase letters show significant differences between the data at the 5% level, using the multiple range test at *p* < 0.05.

**Table 4:** The effect of PGRs on the adventitious shoot induction of *Hypolepis punctata*.

BA	NAA	AC	Sporophyte Regeneration Rate (%)	Average Number of Adventitious Buds per Explant
(mg/L)	(mg/L)	(g/L)		
0.1	0.1	0	72.21 ± 2.94 de	30.32 ± 1.07 e
0.2	0.1	0	81.11 ± 2.22 c	36.29 ± 0.73 d
0.5	0.1	0	90.00 ± 3.33 b	46.10 ± 0.53 b
0.5	0.1	2	98.89 ± 1.11 a	49.19 ± 0.65 a
0.5	0.05	0	81.11 ± 2.94 c	42.11 ± 0.80 c
0.5	0.2	0	86.67 ± 1.93 bc	31.27 ± 1.04 e
1	0.1	0	65.55 ± 2.22 ef	10.41 ± 0.56 gh
1	0.1	2	78.88 ± 2.21 cd	15.52 ± 0.67 f
1	0.5	0	57.78 ± 4.01 fg	12.04 ± 0.25 g
1.5	0.1	0	46.67 ± 3.85 h	9.82 ± 0.58 h
1.5	0.1	2	53.33 ± 1.93 gh	10.33 ± 0.33 gh

Data represent mean ± standard error. Duncan’s various lowercase letters show significant differences between the data at the 5% level, using the multiple range test at *p* < 0.05.

**Table 5:** The effect of BA and AC concentration on the adventitious shoot elongation of *Hypolepis punctata*.

BA (mg/L)	AC (g/L)	Shoot Elongation (%)	Average Length (cm)
0.1	2	60.00 ± 1.92 c	2.51 ± 0.03 d
0.1	0	54.44 ± 1.11 cd	2.30 ± 0.08 d
0.5	2	97.78 ± 2.22 a	4.83 ± 0.19 a
0.5	0	51.11 ± 2.94 d	3.61 ± 0.23 b
1	2	72.22 ± 2.94 b	3.07 ± 0.09 c
1	0	41.11 ± 1.11 e	2.89 ± 0.13 c

Data represent mean ± standard error. Duncan’s various lowercase letters show significant differences between the data at the 5% level, using the multiple range test at *p* < 0.05.

**Table 6:** The effect of IBA concentration on rooting of adventitious shoots.

IBA (mg/L)	Adventitious Root Induction Rate (%)	Number of Roots
0	43.33 ± 1.93 d	6.75 ± 1.64 d
0.3	82.22 ± 2.22 c	15.34 ± 0.62 c
0.5	92.22 ± 2.94 ab	25.27 ± 0.40 a
0.7	98.89 ± 1.11 a	18.91 ± 0.43 b
1.0	88.89 ± 2.94 bc	17.91 ± 2.12 b

Data represent mean ± standard error. Duncan’s various lowercase letters show significant differences between the data at the 5% level, using the multiple range test at *p* < 0.05.

**Table 7:** Detailed amplification results of ISSR primers used in the genetic stability of the in vitro-regenerated plantlets of *Hypolepis punctata*.

Primer	Nucleotide Sequence (5′–3′)	Number of Amplification Band	Size Range of Band (bp)
ISSR3	AGGAGGAGGAGGC	5	100–700 bp
ISSR6	AGAGAGAGAGAGAGAGYT	8	150–500 bp
ISSR8	GAGAGAGAGAGAGAGAG	7	100–400 bp
ISSR10	GAGGAGGAGGAGGC	6	100–600 bp
ISSR11	CTCCTCCTCCTCGG	6	200–1000 bp
ISSR12	CACACACACACAAC	7	200–1000 bp
ISSR13	GGGTGGGGTGGGGTG	5	100–600 bp
ISSR14	GAGAGAGAGAGACC	5	300–700 bp
ISSR15	CAGCAGCAGGC	3	300–600
ISSR20	CCAGAGGAGGAGGAG	3	100–300 bp

## Data Availability

The original contributions presented in this study are included in the article. Further inquiries can be directed to the corresponding authors.

## Abbreviations

GGB, green globular body; BA, 6-benzylaminopurine; NAA, 1-naphthaleneacetic acid; IBA, indole-3-butyric acid; PGR, plant growth regulator; AC, activated charcoal; NaClO, sodium hypochlorite.

## References

[1] Brownsey P.J. (1987). A review of the fern genus *Hypolepis* (Dennstardtiaceae) in the Malesian and Pacific regions. Blumea Biodivers. Evol. Biogeogr. Plants.

[2] Xing F., Wang F., Wu Z., Raven P.H., Hong D. (2013). Hypolepis. Flora of China, Vols. 2–3.

[3] Schwartsburd P.B., Perrie L.R., Brownsey P., Shepherd L.D., Shang H., Barrington D.S., Sundue M.A. (2020). New insights into the evolution of the fern family *Dennstaedtiaceae* from an expanded molecular phylogeny and morphological analysis. Mol. Phylogenet. Evol..

[4] Li L., Yang C.P., Shang H. (2024). Characteristics of intragametophytic selfing of ornamental Ferns *Hypolepis punctata* on sporophyte germination and development. Hortic. Seed.

[5] Huang K., Shang H., Zhou Q., Wang Y., Shen H., Yan Y. (2021). Volatiles Induced from *Hypolepis punctata* (Dennstaedtiaceae) by Herbivores Attract *Sclomina erinacea* (Hemiptera: Reduviidae): Clear Evidence of Indirect Defense in Fern. Insects.

[6] Potter D.M., Baird M.S. (2000). Carcinogenic effects of ptaquiloside in bracken fern and related compounds. Br. J. Cancer.

[7] Hayashi Y., Nishizawa M., Harita S., Sakan T. (1972). Structures and Syntheses of Hypolepin A, B and C, Sesquiterpenes from *Hypolepis punctata* Mett. Chem. Lett..

[8] Lai K. (2003). Studies on the Cytotoxic Principles from *Hypolepis punctata*. Master’s Thesis.

[9] Barnicoat H., Cripps R., Kendon J., Sarasan V. (2011). Conservation in vitro of rare and threatened ferns—Case studies of biodiversity hotspot and island species. In Vitro Cell. Dev. Biol.-Plant.

[10] Liu J.H., Wang Y., Liu B.D. (2012). Observation on gametophyte development of *Hypolepis punctata*. J. Trop. Biol..

[11] Winarto B., da Silva J.A.T. (2012). Improved micropropagation protocol for leatherleaf fern (*Rumohra adiantiformis*) using rhizomes as donor explant. Sci. Hortic..

[12] Lin W., Li Y., Liang J., Liu Y., Chen P., He B., Huang J., Guo L., Lan S. (2024). Establishment of *Dendrobium wilsonii* Rolfe in vitro regeneration system. Sci. Hortic..

[13] Li Y., Yu R.P., Li H., Li D., Shi L. (2012). Research Advances in Tissue Culture of Ornamental Ferns. Acta Hortic. Sin..

[14] Pu Y., Song Q., Wang G., Wu L., Yang C., Yu R. (2023). In vitro propagation and long-term observation of acclimated plants in endangered tree fern *Alsophila costularis*. Plant Cell Tissue Organ Cult. (PCTOC).

[15] Fernández H., Revilla M.A. (2003). In vitro culture of ornamental ferns. Plant Cell Tissue Organ Cult..

[16] Hegde S., Menon V.K., Noronha R., D’Souza L. (2006). Callus culture and an unconventional pattern of sporophyte regeneration in *Drynaria quercifolia*—A medicinal fern. Vitr. Cell. Dev. Biol.-Plant.

[17] Kato Y. (1963). Physiological and morphogenetic studies of fern gametophytes in aseptic culture. *I. Callus* tissues from dark-cultured *Pteris vittata*. Bot. Gaz..

[18] Xiong Y., Zeng Y., Liu J., Chen X., Li Y., Zhang X., Bian Z., da Silva J.A.T., Zeng S., Wu K. (2023). Gametophyte development and sporophyte regeneration of *Alsophila spinulosa*. J. Plant Growth Regul..

[19] Camloha M., Gogala N., Rode J. (1994). Plant regeneration from leaf explants of the fern *Platycerium bifurcatum* in vitro. Sci. Hortic..

[20] Bertrand A.M., Albuerne M.A., Fernandez H., Gonzalez A., Sánchez-Tamés R. (1999). In vitro organogenesis of *Polypodium cambricum*. Plant Cell Tissue Organ Cult..

[21] Higuchi H., Amaki W., Suzuki S. (1987). In vitro propagation of *Nephrolepis cordifolia* Prsel. Sci. Hortic..

[22] Liao Y.K., Wu Y.H. (2011). In vitro propagation of *Platycerium bifurcatum* (cav.) c. chr. via green globular body initiation. Bot. Stud..

[23] Quiroz L.F., Khan M., Gondalia N., Lai L., McKeown P.C., Brychkova G., Spillane C. (2025). Tissue culture-independent approaches to revolutionizing plant transformation and gene editing. Hortic. Res..

[24] Bui L.T., Long H., Irish E.E., Cordle A.R., Cheng C.L. (2018). The power of gametophyte transformation. Current Advances in Fern Research.

[25] Xiang D.L., Li G.S. (2024). Control of leaf development in the water fern *Ceratopteris richardii* by the auxin efflux transporter CrPINMa in the CRISPR/Cas9 analysis. BMC Plant Biol..

[26] Wang F., Zhong Z., Chen L., Shu J., Yan Y. (2024). Overview of gene function research in monilophytes (ferns). J. Integr. Plant Biol..

[27] Chen Z.H. (2022). Unveiling novel genes in fern genomes for the design of stress tolerant crops. Crop Des..

[28] Shukla J.N., Kalsi M., Sethi A., Narva K.E., Fishilevich E., Singh S., Mogilicherla K., Palli S.R. (2016). Reduced stability and intracellular transport of dsRNA contribute to poor RNAi response in lepidopteran insects. RNA Biol..

[29] Murashige T., Skoog F. (1962). A revised medium for rapid growth and bio assays with tobacco tissue cultures. Physiol. Plant..

[30] Chu C.C. (1981). The N6 medium and its applications to anther culture of cereal crops. Proc. Symp. Plant Tissue Cult..

[31] Gupta P.K., Durzan D.J. (1985). Shoot multiplication from mature trees of Douglas-fir (*Pseudotsuga menziesii*) and sugar pine (*Pinus lambertiana*). Plant Cell Rep..

[32] George E.F., Hall M.A., De Klerk G.J. (2007). Plant Propagation by Tissue Culture: Volume 1. The Background (Vol. 1).

[33] Yu R., Li F., Wang G., Ruan J., Wu L., Wu M., Yang C., Shan Q. (2021). In vitro regeneration of the colorful fern Pteris aspericaulis var. tricolor via green globular bodies system. Vitr. Cell. Dev. Biol.-Plant.

[34] Mikuła A., Pożoga M., Grzyb M., Rybczyński J.J. (2015). An unique system of somatic embryogenesis in the tree fern *Cyathea delgadii* Sternb.: The importance of explant type, and physical and chemical factors. Plant Cell Tissue Organ Cult. (PCTOC).

[35] Fernández H., Bertrand A.M., Sanchez-Tames R. (1996). Influence of tissue culture conditions on apogamy in *Dryopteris affinis* sp. affinis. Plant Cell Tissue Organ Cult..

[36] Thakur R.C., Hosoi Y., Ishii K. (1998). Rapid in vitro propagation of *Matteuccia struthiopteris* (L.) Todaro–an edible fern. Plant Cell Rep..

[37] Higuchi H., Amaki W. (1989). Effects of 6-benzylaminopurine on the organogenesis of *Asplenium nidus* L. through in vitro propagation. Sci. Hortic..

[38] Yu R., Zhang G., Li H., Cao H., Mo X., Gui M., Zhou X., Jiang Y., Li S., Wang J. (2017). In vitro propagation of the endangered tree fern *Cibotium barometz* through formation of green globular bodies. Plant Cell Tissue Organ Cult. (PCTOC).

[39] Amaki W., Higuchi H. (1990). A possible propagation system of Nephrolepis, Asplenium, Pteris, Adiantum and Rumohra (Arachniodes) through tissue culture. Vitr. Cult. XXIII IHC.

[40] Cárdenas-Aquino M.D.R., Camas-Reyes A., Valencia-Lozano E., López-Sánchez L., Martínez-Antonio A., Cabrera-Ponce J.L. (2023). The Cytokinins BAP and 2-iP modulate different molecular mechanisms on shoot proliferation and Root Development in Lemongrass (*Cymbopogon citratus*). Plants.

[41] Pan M.J., Staden J.V. (1998). The use of charcoal in in vitro culture–A review. Plant Growth Regul..

[42] Thomas T.D. (2008). The role of activated charcoal in plant tissue culture. Biotechnol. Adv..

[43] Teng W.L. (1997). Activated charcoal affects morphogenesis and enhances sporophyte regeneration during leaf cell suspension culture of *Platycerium bifurcatum*. Plant Cell Rep..

[44] Padhya M.A., Mehta A.R. (1982). Propagation of fern (Nephrolepis) through tissue culture. Plant Cell Rep..

[45] Jang B.K., Cho J.S., Park K., Lee C.H. (2020). Practical methodology for gametophyte proliferation and sporophyte production in green penny fern (*Lemmaphyllum microphyllum* C. Presl) using mechanical fragmentation. Vitr. Cell. Dev. Biol.-Plant.

[46] Yan X., Zheng K., Li P., Zhong X., Zhu Z., Zhou H., Zhu M. (2024). An efficient in vitro organogenesis protocol for the endangered relic tree species *Bretschneidera sinensis* and genetic fidelity assessment using DNA markers. Front. Plant Sci..

[47] Nalousi A.M., Hatamzadeh A., Azadi P., Mohsenpour M., Lahiji H.S. (2019). A procedure for indirect shoot organogenesis of *Polianthes tuberosa* L. and analysis of genetic stability using ISSR markers in regenerated plants. Sci. Hortic..

[48] Zhou H., Sun J., Zheng K., Zhang X., Yao Y., Zhu M. (2024). Efficient Plantlet Regeneration from Branches in *Mangifera indica* L. Plants.

[49] Shang H., Wang Y., Yan Y.H. (2015). Development and characterization of microsatellite loci in the pantropical fern *Hypolepis punctata* (Dennstaedtiaceae). Appl. Plant Sci..

